# SnoopLigase peptide-peptide conjugation enables modular vaccine assembly

**DOI:** 10.1038/s41598-019-40985-w

**Published:** 2019-03-15

**Authors:** Anne-Marie C. Andersson, Can M. Buldun, David J. Pattinson, Simon J. Draper, Mark Howarth

**Affiliations:** 1 Department of Biochemistry, University of Oxford, South Parks Road, Oxford, OX1 3QU UK; 2 Jenner Institute, University of Oxford, Oxford, OX3 7DQ UK

## Abstract

For many infectious diseases there is still no vaccine, even though potential protective antigens have been identified. Suitable platforms and conjugation routes are urgently needed to convert the promise of such antigens into broadly protective and scalable vaccines. Here we apply a newly established peptide-peptide ligation approach, SnoopLigase, for specific and irreversible coupling of antigens onto an oligomerization platform. SnoopLigase was engineered from a *Streptococcus pneumoniae* adhesin and enables isopeptide bond formation between two peptide tags: DogTag and SnoopTagJr. We expressed in bacteria DogTag linked to the self-assembling coiled-coil nanoparticle IMX313. This platform was stable over months at 37 °C when lyophilized, remaining reactive even after boiling. IMX-DogTag was efficiently coupled to two blood-stage malarial proteins (from PfEMP1 or CyRPA), with SnoopTagJr fused at the N- or C-terminus. We also showed SnoopLigase-mediated coupling of a telomerase peptide relevant to cancer immunotherapy. SnoopLigase-mediated nanoassembly enhanced the antibody response to both malaria antigens in a prime-boost model. Including or depleting SnoopLigase from the conjugate had little effect on the antibody response to the malarial antigens. SnoopLigase decoration represents a promising and accessible strategy for modular plug-and-display vaccine assembly, as well as providing opportunities for robust nanoconstruction in synthetic biology.

## Introduction

Highly successful vaccines, such as against smallpox or polio, mediate protection primarily via antibodies targeting low variability antigens^1^. Diseases resistant to vaccination, such as malaria or HIV, show high antigen sequence diversity and transient antigen availability for targeting by the immune system^2–5^. Such characteristics present a great challenge when vaccine efficacy requires high serum antibody titers, combined with long-lived antibody responses^6^. Outstanding examples where such responses have been induced are the polio^7^, yellow fever^8^, measles/mumps/rubella^9^, and the Human Papilloma Virus (HPV) vaccines^10^. Only a single immunization is required for Cervarix and Gardasil to induce long-lasting protective antibody responses^11,12^. These virus-like particle (VLP) vaccines present the antigen in dense arrays on a multimerizing scaffold. The great enhancement of immune responses via multimeric antigen display has been validated for numerous antigens^13–15^. The high density and suitable spacing of antigen on the surface of the scaffold leads to B cell receptor cross-linking and promotes strong activation of the B cells^16,17^, initiating germinal center reaction, a prerequisite for the development of high affinity antibodies^18^. In addition, the size of VLPs promotes lymph node homing and uptake by antigen presenting cells for processing into peptides for presentation on MHC class II for CD4^+^ T cell activation^19^.

Apart from using VLPs, fusion of antigens to oligomerizing proteins such as the non-structural protein of rotavirus (NSP4)^20^ or flagellin^21,22^ had a beneficial effect on immune responses. Another notable scaffold is the complement inhibitor C4-binding protein (C4bp)^23^. A single domain is necessary for C4bp to form self-assembling heptamers. A hybrid domain of chicken C4bp was used to generate IMX313 (here abbreviated to IMX)^23^. Such a platform gave enhanced responses to blood-stage malaria antigen Merozoite Surface Protein-1 (MSP-1) and the transmission-blocking malaria antigen Pfs25^23–25^. Pfs25-IMX is currently being considered as part of a multi-vaccine approach and has recently been tested in a phase I clinical trial (NCT02532049)^26^.

Chimeric oligomeric assemblies can be generated by direct genetic fusion of the antigen of interest to the oligomerizing protein unit^25,27–29^. Major challenges frequently found with such genetic fusions are: (1) misfolding of the antigen; (2) disrupted assembly of the carrier; and (3) size-limits on the antigen for successful fusion^30,31^. Post-translational approaches provide an important alternative way to connect a display platform to an antigen. These methods include click chemistry^32^, sortase-mediated attachment^33,34^, affinity tag conjugations^35,36^, Ni-NTA:His-tagged interaction^37^, and chemical cross-linking^38,39^. Such approaches have faced a number of fundamental challenges regarding stability, scalable production and specificity of coupling, as recently reviewed^31^.

In previous work, we showed efficient modular oligomerization of antigens for vaccine assembly using a “Plug-and-Display” platform. SpyCatcher was genetically fused to the coat protein of the AP205 bacteriophage for assembly into VLPs (Supplementary Fig. S1a). Mixing with SpyTag-fused antigen allowed efficient and irreversible conjugation to the VLPs via spontaneous isopeptide bond formation (Supplementary Fig. S1b)^13^. We further established a dual “plug-and-display” platform on the IMX scaffold, taking advantage of the SnoopTag/SnoopCatcher covalent interaction (Supplementary Fig. S1c,d). IMX was fused at the N-terminus to SpyCatcher and at the C-terminus to SnoopCatcher, allowing oligomerization of both SpyTag-fused antigen and SnoopTag-fused antigen (Supplementary Fig. S1c)^14^. However, in these previous examples, the Tag/Catcher conjugation depends on fusing one partner to a Catcher protein of at least 80 residues^13,14^. Therefore, to minimize the size of fusion required, we have now developed SnoopLigase for efficient peptide-peptide ligation^40^. SnoopLigase can achieve high yielding and specific ligation of two peptides in a range of protein locations under mild conditions with as low as 2.5 µM concentration of substrate^40^. Here we explore the potential of SnoopLigase for nanoassembly of modular vaccines, using malaria antigens for proof-of-concept.

## Results

### IMX-DogTag expression and reactivity

**Figure 1:**
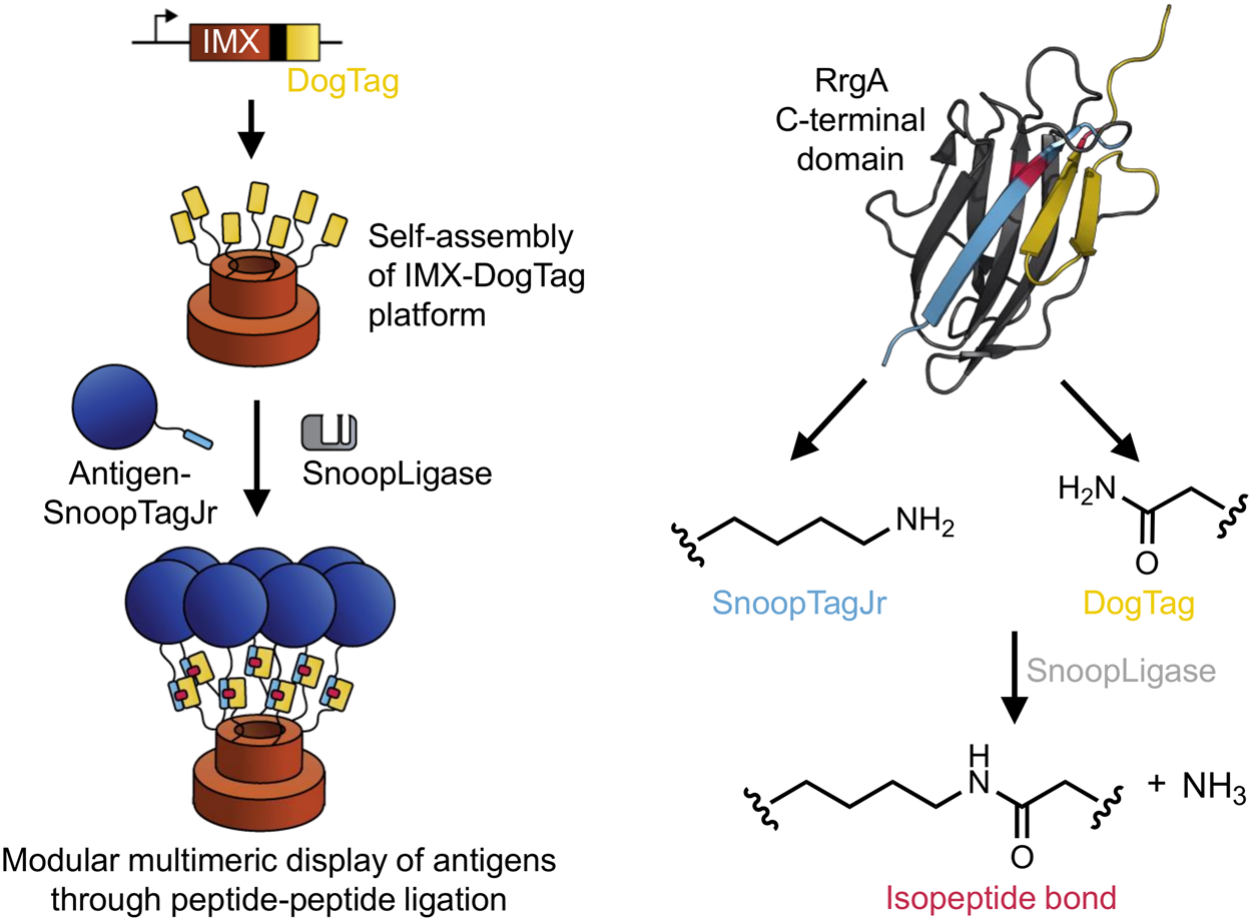
Overview of SnoopLigase-mediated vaccine assembly. IMX is fused to DogTag at its C-terminus. When expressed in *E*. *coli*, spontaneous oligomerization yields IMX−DogTag nanoparticles. SnoopLigase promotes isopeptide bond formation upon mixing of IMX-DogTag with antigen-SnoopTagJr. The RrgA C-terminal domain (Protein Data Bank 2WW8) was previously split into three parts and engineered. The reactive Lys is located on SnoopTagJr (cyan), the reactive Asp on DogTag (yellow), and the catalytic Glu on SnoopLigase (gray) (key residues are highlighted in red).

We genetically fused the DogTag peptide to the C-terminus of IMX^23,26,41^. IMX forms primarily heptamers stabilized by interchain disulfide bonds^23^. Then our strategy was to fuse genetically our antigen of interest to the SnoopTagJr peptide. Adding SnoopLigase would lead to covalent coupling of DogTag to SnoopTagJr through isopeptide bond formation, enabling modular oligomerization of the antigen (Fig. 1).

**Figure 2:**
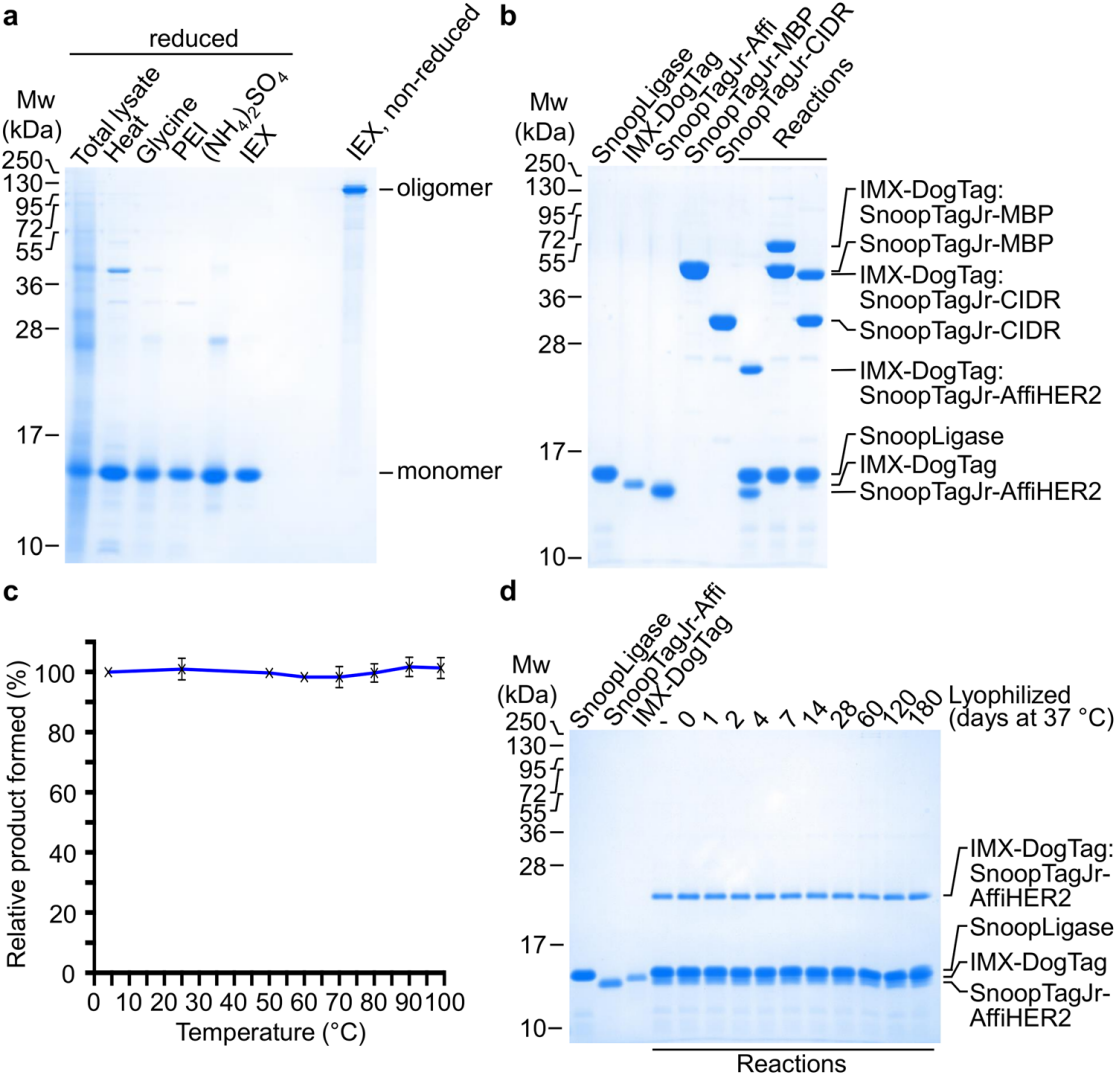
IMX-DogTag purification and resilience to heat and lyophilization. (**a**) Nanoparticle purification. IMX−DogTag was induced in *E*. *coli* (total lysate). Protein was purified by treatment with heat, glycine, PEI, (NH_4_)_2_SO_4_, and a final ion exchange (IEX) step. Samples were analyzed by SDS-PAGE and Coomassie staining under reducing conditions except where indicated. (**b**) IMX-DogTag conjugation with indicated proteins. SnoopTagJr-AffiHER2, SnoopTagJr-MBP or SnoopTagJr-CIDR at 20 μM were conjugated to 10 μM IMX-DogTag using 20 μM SnoopLigase for 48 h at 4 °C. Samples were boiled in the presence of reducing agent, and analyzed by SDS-PAGE and Coomassie staining. (**c**) IMX-DogTag reactivity after exposure to different temperatures. IMX-DogTag was treated for 2 h at the indicated temperature and cooled for 30 min at 12 °C. Heat-treated IMX-DogTag was reacted with SnoopTagJr-AffiHER2 with each protein at 10 μM for 2 h at 4 °C. Reaction was analyzed by SDS-PAGE with Coomassie staining and the unheated sample set at 100% (mean of triplicate ± s.d.; some error bars are too small to be visible). (**d**) IMX-DogTag reactivity after storing lyophilized at 37 °C. IMX-DogTag was lyophilized and incubated for the indicated days at 37 °C. IMX-DogTag was then reacted with SnoopTagJr-AffiHER2 and SnoopLigase for 2 h at 4 °C, before analyzing under reducing conditions by SDS-PAGE with Coomassie staining.

IMX-DogTag was expressed to a high level in *Escherichia coli* (Fig. 2a). To avoid having a tag for affinity purification which would itself raise antibodies upon immunization (e.g. His_6_)^42^, we took advantage of the extreme thermostability of the IMX-DogTag particle. We incubated cell lysate at 80 °C for 15 min, which led to aggregation of most endogenous proteins but IMX-DogTag remained in solution (Fig. 2a). To achieve high purity, we performed sequential precipitations with low pH, polyethyleneimine (PEI) (to remove any DNA-binding proteins), and ammonium sulfate, followed by anion exchange chromatography (the pI of IMX-DogTag is 5.1). To promote disulfide bond formation, IMX-DogTag was incubated with the oxidizing agent diamide. Non-reducing sodium dodecyl sulfate polyacrylamide gel electrophoresis (SDS-PAGE) revealed the formation of a high molecular weight species of IMX-DogTag, as expected^23,41^ (Fig. 2a). The final IMX-DogTag yield was at least 10 mg per liter of shake-flask culture. IMX-DogTag at 10 μM was coupled by SnoopLigase at 20 μM to three different SnoopTagJr-linked proteins: an affibody to HER2 (AffiHER2), maltose binding protein (MBP) and the Cysteine-rich Inter-domain Region (CIDR), all at 20 μM. CIDR is one of the extracellular domains of *Plasmodium falciparum* Erythrocyte Membrane Protein 1 (PfEMP1), which is involved in the interaction between infected red blood cells and endothelial cells^43^. The binding of CIDR to the endothelial protein receptor C has further been associated with severe malaria^44^. We validated efficient covalent reaction of IMX-DogTag with each of the antigens by the appearance of a band of the expected molecular weight that was resistant to boiling in SDS (Fig. 2b)

### IMX-DogTag is highly resilient

To analyze the robustness of the IMX-DogTag scaffold, IMX-DogTag was incubated at various temperatures from 4 °C to 99 °C and then incubated with SnoopTagJr-AffiHER2 and SnoopLigase for 2 h at 4 °C, all at 10 μM. The relative product formation was estimated by SDS-PAGE. Even following 99 °C incubation of IMX-DogTag, we observed no loss of reactivity for SnoopLigase coupling (Fig. 2c). IMX-DogTag reactivity was also investigated after lyophilization of the scaffold and storage at 37 °C for up to 180 days. 10 μM IMX-DogTag was reacted with 10 μM SnoopTagJr-AffiHER2 and 20 μM SnoopLigase for 2 h at 4 °C, and analyzed by SDS-PAGE. No change in product formation was observed after storage of lyophilized IMX-DogTag at 37 °C for 180 days (Fig. 2d).

### Expression and oligomerization of malarial antigens and peptide antigen

**Figure 3:**
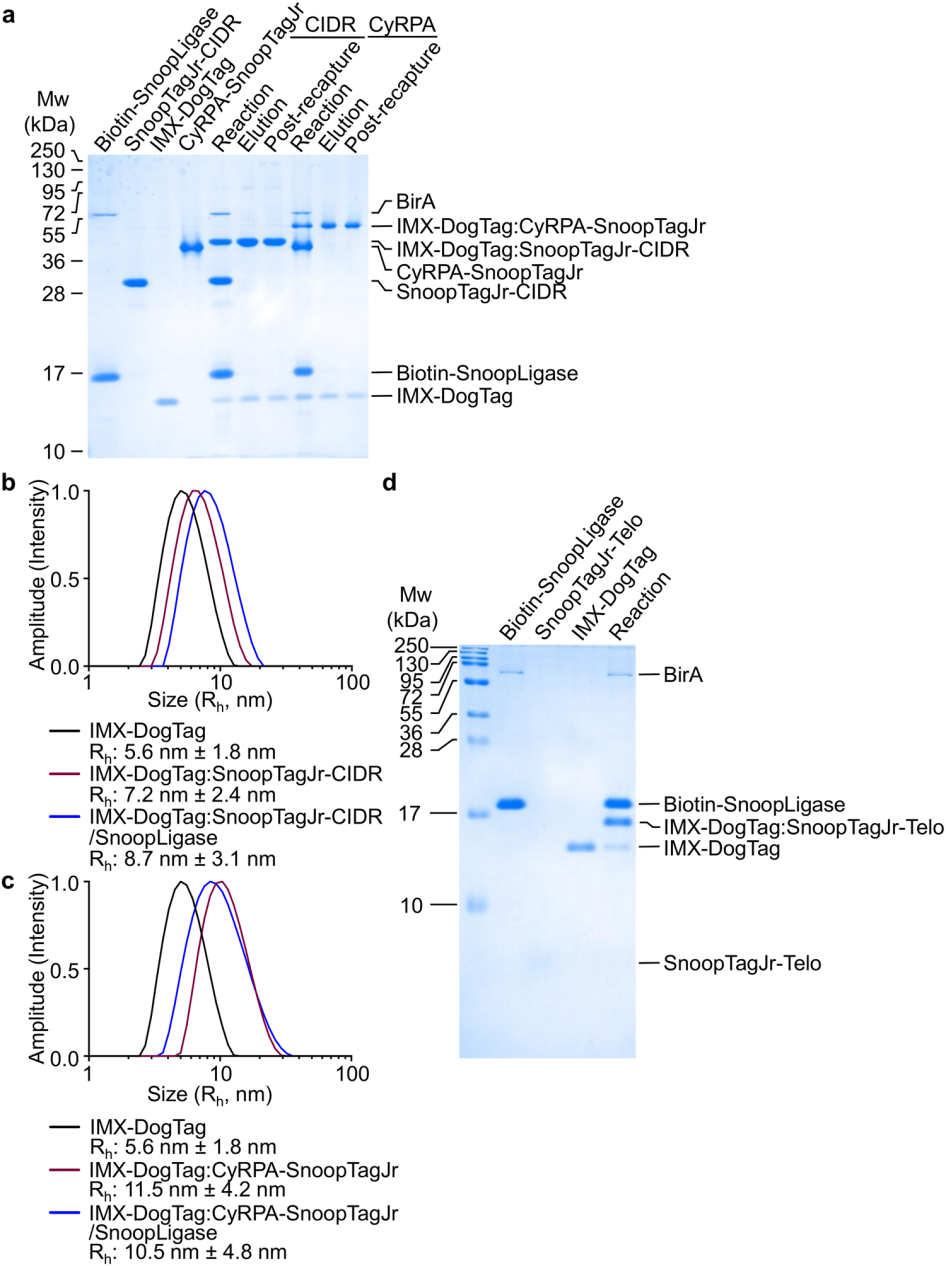
IMX-DogTag conjugation to antigens. (**a**) Efficient covalent coupling to the nanoparticle by SnoopLigase. IMX-DogTag was incubated with 1.5-fold molar excess of SnoopTagJr−CIDR or CyRPA-SnoopTagJr for 48 h at 4 °C. Biotin-SnoopLigase was removed efficiently from IMX-DogTag:Antigen-SnoopTagJr conjugates after elution from Streptavidin-agarose, or a second round of Streptavidin-agarose purification (post-recapture). Samples were analyzed by reducing SDS-PAGE with Coomassie staining. (**b**) Hydrodynamic radius of IMX-DogTag before and after coupling to CIDR. The hydrodynamic radius (R_h_) was determined by DLS for unconjugated IMX-DogTag and IMX-DogTag conjugated with SnoopTagJr-CIDR, with or without SnoopLigase removal. (**c**) Hydrodynamic radius of IMX-DogTag before and after coupling to CyRPA, as in (b). **(d)** IMX-DogTag reactivity with SnoopTagJr-Telo peptide. IMX-DogTag was reacted with SnoopTagJr-Telo in the presence of biotinylated SnoopLigase for 48 h at 4 °C and analyzed under reducing conditions by SDS-PAGE with Coomassie staining.

Two malarial proteins were used to investigate the effect of their oligomerization by IMX-DogTag on immunogenicity. SnoopTagJr-CIDR, as above, had the tag fused to its N-terminus. In addition, we tested reaction with IMX-DogTag of the blood-stage antigen Cysteine-Rich Protective Antigen (CyRPA), a part of a multi-protein complex necessary for *P*. *falciparum* merozoite invasion of red blood cells^45^. The high conservation of CyRPA within *P*. *falciparum* isolates^46^ and the discovery of PfCyRPA-specific monoclonal antibodies that can prevent red blood cell invasion^47^ makes CyRPA a promising vaccine target. SnoopTagJr was genetically fused to the C-terminus of CyRPA and CyRPA-SnoopTagJr was expressed in mammalian cells. The antigen was purified using C-tag affinity resin and size-exclusion chromatography. Conjugation of CyRPA-SnoopTagJr to IMX-DogTag mediated by SnoopLigase was validated by SDS-PAGE (Fig. 3a).

For immune response analysis, we were interested to test the effect of the presence or absence of SnoopLigase in the vaccine formulation. SnoopLigase was originally engineered by splitting a *Streptococcus pneumoniae* domain into three fragments. This design process meant that, after directing reaction of DogTag with SnoopTagJr, SnoopLigase remains stably associated with the ligated product^40^. We had previously developed a method for removing SnoopLigase site-specifically biotinylated by BirA from SnoopTagJr:DogTag conjugates after ligation^40^. This method was adapted for SnoopLigase removal from IMX-DogTag conjugates. After reacting IMX-DogTag with SnoopTagJr-fused antigen, biotin-SnoopLigase was captured with streptavidin-agarose. Sequential washes were performed with 1 M imidazole to remove excess unreacted antigen. To elute the IMX-DogTag conjugate, the resin was incubated with 3.5 M imidazole. This elution condition enabled the release of the IMX-DogTag conjugate from biotin-SnoopLigase with occasionally a trace of biotin-SnoopLigase coming off the resin. To ensure even further removal of biotin-SnoopLigase from the IMX-DogTag conjugate, the eluted product was re-incubated with fresh streptavidin-agarose (Fig. 3a). The purified heptamer also included some unreacted IMX-DogTag, indicating that coupling to these antigens did not go to completion (Fig. 3a).

Analysis of the IMX-DogTag by dynamic light scattering (DLS) revealed a hydrodynamic radius of 5.6 ± 1.8 nm (Fig. 3b). There was an increase in the hydrodynamic radius when conjugated to SnoopTagJr-fused antigens (Fig. 3b,c). The hydrodynamic radius increased with SnoopLigase present on the IMX-DogTag:SnoopTagJr-CIDR conjugate (from 7.2 ± 2.4 nm to 8.7 ± 3.1 nm), whereas a slight decrease in the hydrodynamic radius was observed for the IMX-DogTag:CyRPA-SnoopTagJr conjugate with SnoopLigase (from 11.5 ± 4.2 nm to 10.5 ± 4.8 nm) (Fig. 3b,c). This difference may relate to an alteration in how CyRPA projects away from the nanoparticle.

Modular decoration of vaccine platforms also has potential in generation of cancer vaccines, given the powerful responses achieved against patient-specific neoantigens^31,48^. We also established the conjugation of a cancer-relevant peptide by SnoopLigase to IMX-DogTag. Telo (16 amino acids) is derived from a human telomerase reverse transcriptase (hTERT) mutant epitope; hTERT is overexpressed in >85% of human cancers^49^. We observed 78% conjugation of IMX-DogTag, after incubating with SnoopTagJr-Telo (30 aa, 3.3 kDa) and SnoopLigase (Fig. 3d).

### Immunization using IMX-DogTag

**Figure 4:**
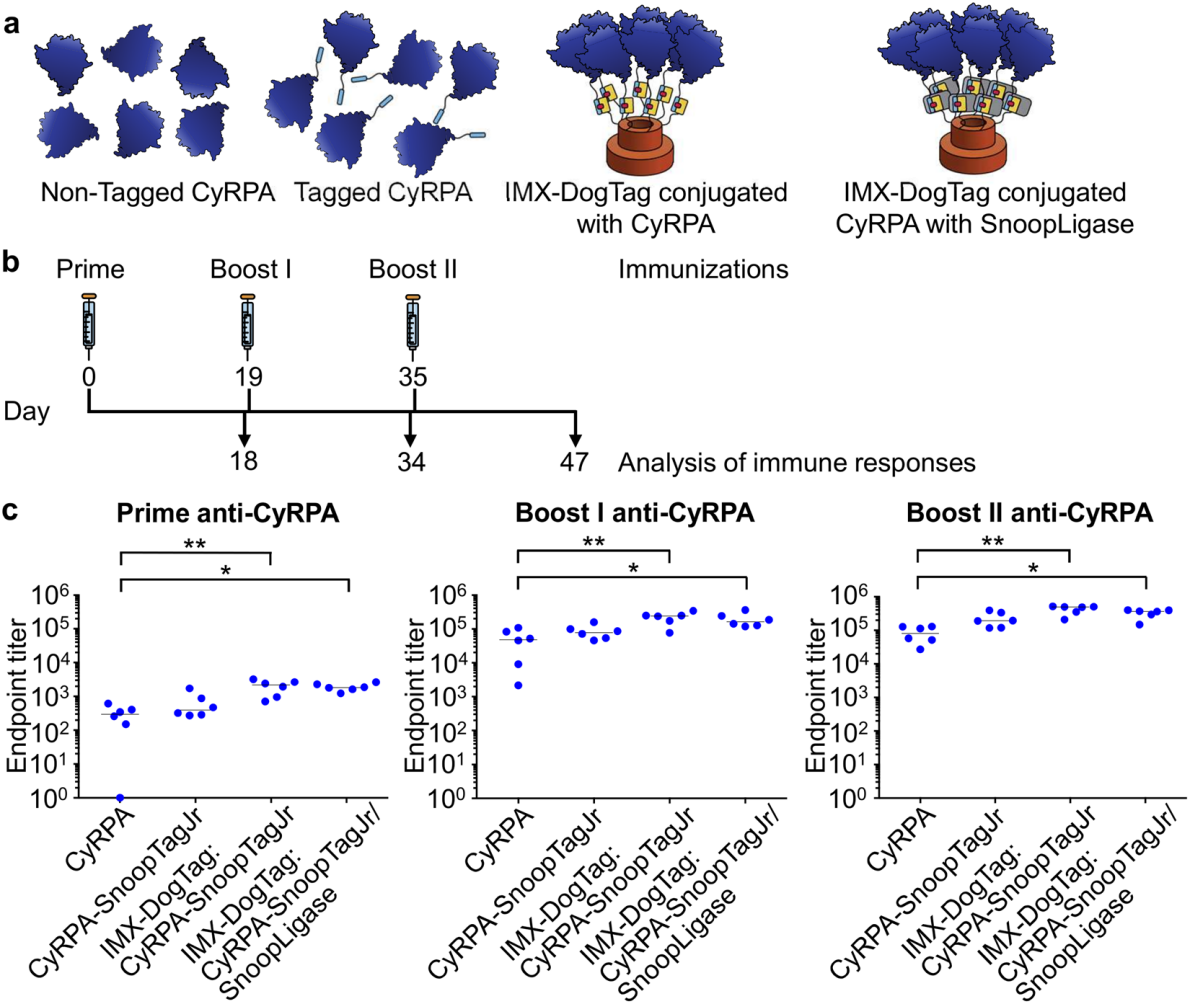
Nanoparticle immunization against CyRPA. (**a**) Schematic of the different immunogens used. (**b**) Schematic of time-points of immunization and analysis of immune responses. (**c**) CyRPA-specific IgG responses after nanoparticle immunization. Mice immunized with either IMX-DogTag:CyRPA-SnoopTagJr or IMX-DogTag:CyRPA-SnoopTagJr/SnoopLigase nanoparticles, CyRPA-SnoopTagJr or CyRPA (6 mice per group) at day 0, 19 and 35, were analyzed for CyRPA-specific responses at prime, boost I, and boost II by ELISA. Each dot represents an individual mouse. The horizontal line is the median. ^*^p < 0.05 and ^**^p < 0.01, determined using Kruskal-Wallis test (n = 6) with Dunn’s multiple comparison post-test.

We investigated the impact of antigen oligomerization from IMX peptide-peptide ligation by immunization of mice with malarial antigens. We first immunized mice intramuscularly with 1 μg equivalent of CyRPA either untagged, fused to SnoopTagJr, as an IMX-DogTag:CyRPA-SnoopTagJr conjugate, or as an IMX-DogTag:CyRPA-SnoopTagJr/SnoopLigase conjugate (Fig. 4a) (i.e. without SnoopLigase removal). All vaccines were formulated 1:1 in AddaVax, a squalene-based oil-in-water nano-emulsion adjuvant with a formulation similar to the licensed MF59 adjuvant^50^. Mice were immunized three times with the same vaccine, with boosts performed on day 19 and 35 (Fig. 4b). Antigen-specific total IgG responses were determined by enzyme-linked immunosorbent assay (ELISA). CyRPA-specific antibody responses were detected in all four groups after the priming immunization (Fig. 4c). After boost I, both IMX-DogTag-conjugate vaccines induced a significantly higher IgG response compared to CyRPA alone (IMX-DogTag: CyRPA-SnoopTagJr p = 0.0065; IMX-DogTag:CyRPA-SnoopTagJr/SnoopLigase p = 0.0132) (Kruskal–Wallis test with Dunn’s multiple comparison, n = 6) (Fig. 4c).

After boost II, the antibody responses were again significantly higher for IMX-DogTag conjugated CyRPA compared to CyRPA alone (IMX-DogTag:CyRPA-SnoopTagJr boost I p = 0.0087; boost II p = 0.0010); IMX-DogTag:CyRPA-SnoopTagJr/SnoopLigase post first boost p = 0.0197; post second boost p = 0.0291) (Kruskal–Wallis test with Dunn’s multiple comparison, n = 6). All four groups increased by approximately 2 logs from the priming to the first boost immunization. The CyRPA-specific responses seemed to plateau after the first boost, with less than 0.5 log increase in responses after the second booster immunization (Fig. 4c).

**Figure 5:**
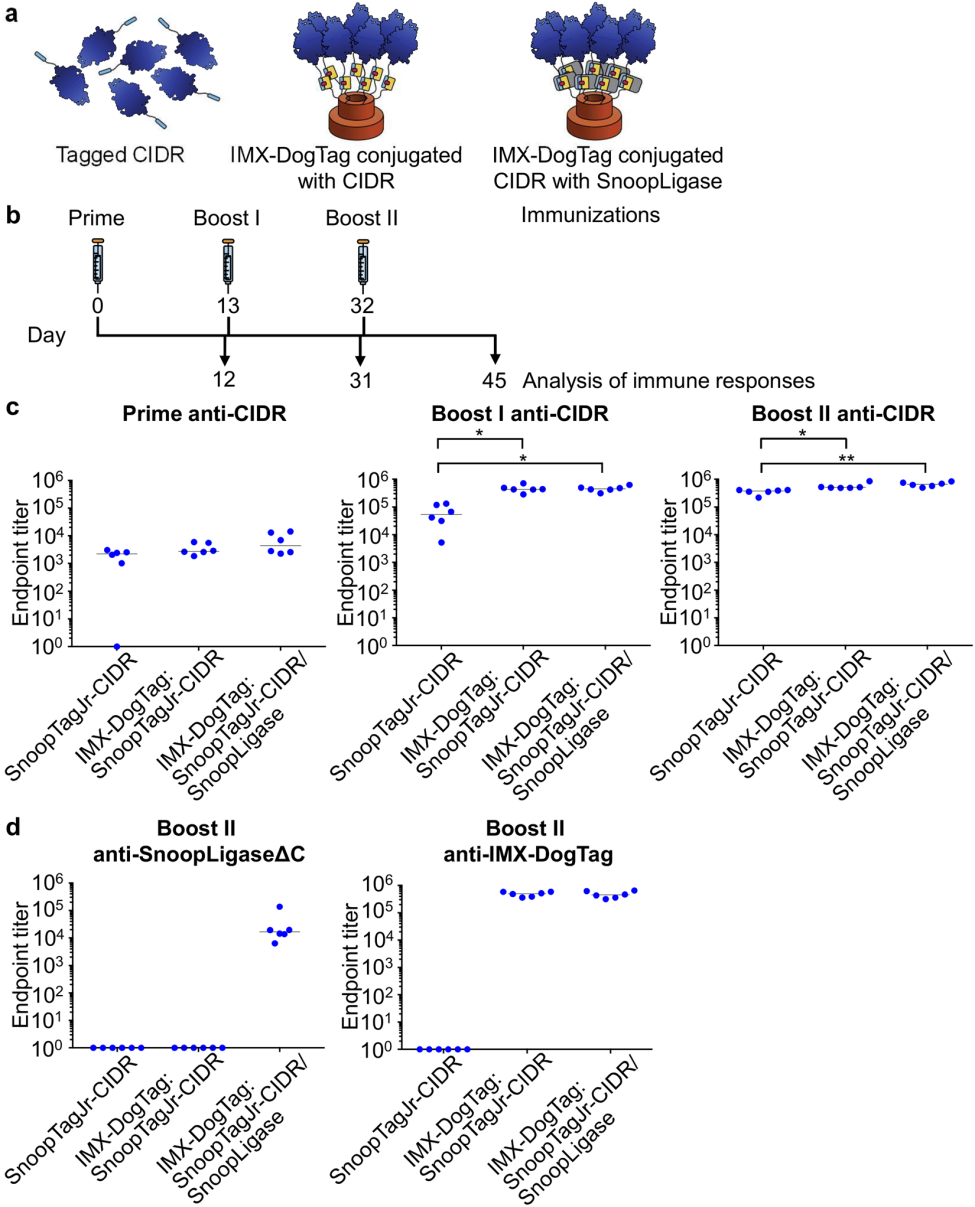
Nanoparticle immunization against CIDR. (**a**) Schematic of the different immunogens used. (**b**) Schematic of time-points of immunization and analysis of immune responses. (**c**) CIDR-specific IgG responses after nanoparticle immunization. Mice immunized with either IMX-DogTag:SnoopTagJr-CIDR or IMX-DogTag:SnoopTagJr-CIDR/SnoopLigase nanoparticles or SnoopTagJr-CIDR (6 mice per group) at day 0, 13 and 32, were analyzed for CIDR-specific responses after prime, boost I, and boost II by ELISA. (**d**) Anti-platform response after immunization. Mice were immunized as in (**c**) and the IgG response to SnoopLigaseΔC or IMX-DogTag was determined by ELISA. Each dot represents an individual mouse. The horizontal line is the median. ^*^p < 0.05 and ^**^p < 0.01, determined using Kruskal-Wallis test (n = 6) with Dunn’s multiple comparison post-test.

To investigate the effect of SnoopLigase-mediated oligomerization with a different antigen, mice were immunized intramuscularly with 1 μg equivalent of CIDR fused to SnoopTagJr on its own, as an IMX-DogTag:SnoopTagJr-CIDR conjugate, or as an IMX-DogTag:SnoopTagJr-CIDR/SnoopLigase conjugate (Fig. 5a). The vaccines were formulated 1:1 in AddaVax. Mice were boosted on day 13 and 32 with the same vaccine regimen (Fig. 5b). The antibody titers against CIDR were determined via ELISA (Fig. 5c). Each vaccine induced similar anti-CIDR IgG responses after the priming immunization, although one mouse failed to respond to monomeric antigen. After the first and the second boost, significantly higher antibody titers compared to SnoopTagJr-CIDR were induced by both IMX-DogTag:SnoopTagJr-CIDR/SnoopLigase (boost I p = 0.0105; boost II p = 0.0020) and IMX-DogTag:SnoopTagJr-CIDR (boost I p = 0.0105; boost II p = 0.0449) (Kruskal–Wallis test with Dunn’s multiple comparison, n = 6). CIDR-specific immune responses only rose slightly in IMX-DogTag-conjugate groups between the first and second boost, while the antibody response rose by approximately 7-fold in the monomeric SnoopTagJr-CIDR immunized group (Fig. 5c).

### Immune responses against IMX-DogTag or SnoopLigase

To understand the immune response generated to the vaccination platform, we analyzed the anti-SnoopLigase and anti-IMX-DogTag antibody responses after the second boost. Since SnoopLigase contains residues also present in DogTag, we generated a shorter version of SnoopLigase, SnoopLigaseΔC, to remove any cross-reactivity from antibodies that might recognize the 7 overlapping residues to DogTag (Supplementary Fig. S2). Only the groups where SnoopLigase had not been removed showed a detectable IgG antibody response against SnoopLigaseΔC (Fig. 5d). This is consistent with our efficient depletion of biotin-SnoopLigase from the conjugate using streptavidin-agarose (Fig. 3a). As expected the IMX-DogTag specific antibody response was high in both IMX-DogTag conjugate vaccinated groups, indicating that neither antigen nor SnoopLigase completely shielded the IMX-DogTag nanoparticle (Fig. 5d). An equivalent pattern of anti-platform response was seen in the anti-CyRPA immunizations (Supplementary Fig. S3).

## Discussion

Here we have established a platform for antigen oligomerization utilizing peptide-peptide conjugation. SnoopLigase allows covalent conjugation of complex protein or synthetic peptide antigens to the IMX-DogTag nanoparticle platform. For a vaccine platform to be scalable, i.e. having the potential for use in vaccination world-wide, a simple production route and stability to sub-optimal storage are important characteristics^30,31^. IMX-DogTag can be purified solubly from *E*. *coli* without any purification tag. IMX-DogTag also showed strong resilience to storage over 6 months and temperatures up to 99 °C. SnoopLigase conjugation generated nanoparticles that induced high-titer antibody responses against two different malarial proteins.

The malaria vaccine nearest to licensure, RTS,S/AS01, is a chimeric VLP targeting a liver-stage antigen. RTS,S/AS01 faces limitations in rapidly waning efficacy^51^, some safety concerns^52^, and the need for four separate doses^53^, bringing additional cost and risk of non-compliance^54^. Immunization to other life-cycle stages is likely to be important for the highest vaccine efficacy^5^. It was previously found that the monomeric blood-stage antigen CyRPA was highly immunogenic^47,55^. Oligomerization of CyRPA on IMX-DogTag improved the IgG response significantly compared to monomeric CyRPA. We also found that conjugation of CIDR to IMX-DogTag significantly improved immunogenicity. In each case, the difference in response between monomer and oligomer was greatest after the first boost, as might be the preferred regimen in human vaccines, since more than two immunizations increases cost and complexity^56^.

DogTag is a smaller fusion partner than ΔN1-SpyCatcher (23 versus 92 residues). DogTag has previously been successfully fused in loops of folded proteins^40^, where SpyCatcher is likely to be less well tolerated^57^. Smaller fusions may also reduce the potential anti-platform immune response. However, it is uncertain if antibody responses induced to SpyCatcher on VLPs negatively impact the immune response to the linked antigen^58–61^. We could clearly detect antibodies to the IMX-DogTag platform, as well as to the SnoopLigase moiety, if SnoopLigase was not depleted. Interestingly, depletion of SnoopLigase had no significant effect on the immune response to the malaria antigens, suggesting that this step may not be required.

Coupling a DogTag-linked peptide rather than a protein to the vaccine platform should also allow antigen to be generated by solid-phase peptide synthesis, which is faster and cheaper for Good Manufacturing Practice (GMP) production. Personalized vaccines^62^ have great potential for immunization against neoepitopes in cancer immunotherapy^48^ and will be important to explore in future studies.

SpyTag/SpyCatcher coupling has been used to decorate a range of immunization platforms (VLPs^13,15,24,63–65^, viruses^66^ and bacterial vesicles^67^) with diverse antigens (relating to parasites, viruses, bacteria, cancer and autoimmunity)^31^. We hope that the SnoopLigase conjugation validated here will show similar broad application. Peptide-peptide coupling to nanoparticles should also provide new opportunities to engineer catalytic nanocompartments^68–72^ and control cellular behavior^73,74^.

## Methods

### Cloning

All constructs were cloned using standard PCR methods and Gibson isothermal assembly. Inserts were verified by Sanger sequencing. Constructs for expression in *E*. *coli* contained an N-terminal His_6_-tag, except for IMX-DogTag. pET28a-SnoopTagJr-AffiHER2, pET28a-SnoopLigase (Supplementary Fig. S1, GenBank Accession No. MG867372) and pET28a-AviTag-SnoopLigase (Addgene plasmid ID 105626) have been published^40^. pET28a-SnoopLigaseΔC is SnoopLigase with 7 amino acids removed from the C-terminus (Supplementary Fig. S2, GenBank Accession No. MH798874). pET28a-IMX-DogTag (Supplementary Fig. S2, GenBank Accession No. MH798875 and Addgene ID 113765) has the following organization: IMX313, GSGSGEGSG spacer, DogTag. CIDR constructs have the following organization: pET15b-SnoopTagJr-CIDR(IT4var07) (GenBank Accession No. MH798877): His_6_, TEV site, GS spacer, SnoopTagJr, GGS spacer, CIDR; pET15b-CIDR(IT4var07): His_6_, TEV site, GGS spacer, CIDR. pENTR4-LPTOS-CyRPA-SnoopTagJr (GenBank Accession No. MH798876) has the organization: IgG leader of V-kappa sequence, *P*. *falciparum* CyRPA (3D7), (GSG)_3_ spacer, SnoopTagJr, GSG spacer, C-Tag. pENTR4-LPTOS-CyRPA has the organization: IgG leader of V-kappa sequence, CyRPA, GGGS spacer, C-Tag. CyRPA constructs contained mutations to remove N-linked glycosylation sites at residues 139, 316, and 332.

The TEV protease vector was a kind gift from Stephen Bottomley, Monash University, and has the organization: MBP, His_6_, TEV protease, Arg_5_. The constructs pGEX-GST-BirA, pET28a-SnoopTagJr-MBP (Addgene plasmid ID 105628 and GenBank Accession No. MG867374) and pET28a-SnoopTagJr-AffiHER2 and their expression have been described previously^40,75^. Molecular weights were predicted from the amino acid sequence by ExPASy ProtParam: SnoopLigase 13.3 kDa; AviTag-SnoopLigase 15.3 kDa; SnoopLigaseΔC 13.3 kDa; IMX-DogTag 9.7 kDa monomer and 67.9 kDa heptamer; SnoopTagJr-AffiHER2 11.7 kDa; SnoopTagJr-CIDR(IT4var07) 29.8 kDa; CIDR(IT4var07) 28.3 kDa; CyRPA-SnoopTagJr 42.0 kDa; CyRPA 40.1 kDa; SnoopTagJr-MBP 43.9 kDa; GST-BirA 61.3 kDa; MBP-TEV protease 71.3 kDa. IMX-DogTag ran higher than its M_w_ on SDS-PAGE, which is consistent with the low mobility of proteins with a high fraction of Asp and Glu^76^.

### Expression and purification of IMX-DogTag

*E*. *coli* BL21 (DE3) RIPL expressing phosphogluconolactonase, to degrade 6-phosphogluconolactone which promotes protein gluconylation^77^, were transformed with pET28a-IMX-DogTag and grown for 16 h at 37 °C. Single colonies were picked and grown in LB with 100 μg/mL ampicillin for 16 h at 37 °C, 200 rpm. The cultures were diluted 1:100 in pre-warmed LB and grown at 37 °C until A_600_ reached 0.25. The cultures were then grown at 25 °C until A_600_ reached 0.6 and induced with 0.42 mM isopropyl β-D-1-thiogalactopyranoside (IPTG) for 4 h. Cells were lysed in 25 mM Tris-phosphate, 25 mM citric acid, pH 5.0 with 10 mM phenylmethylsulfonylfluoride (PMSF) (Sigma) and EDTA-free mixed protease inhibitors (Roche). Cells were sonicated at 50% duty cycle 6 times for 1 min with 1 min break in between. The sonicated cell lysate was then incubated at 80 °C for 15 min, then cooled down at 4 °C for 5 min. Following incubation with 100 mM 2-mercaptoethanol for 5 min at 25 °C, lysates were centrifuged at 30,000 *g* at 4 °C for 30 min. The supernatant was collected and 3 mL 500 mM glycine pH 2.0 was added per 10 mL supernatant. After centrifugation for 30 min at 30,000 g, 4 °C, the pellets were resuspended in 50 mM Tris pH 8.0 containing 2 mM dithiothreitol (DTT). Polyethyleneimine (PEI) (Sigma-Aldrich) was added to a final concentration of 0.1% and supernatants incubated for 1 min at 25 °C. Following centrifugation for 30 min at 30,000 *g* and 4 °C, the supernatant was harvested and ammonium sulfate was added to a final concentration of 2.25 M. After a final centrifugation for 30 min at 30,000 *g* at 4 °C, the pellets were resuspended in 10 mM Tris•HCl pH 8.0 and then dialyzed 1:1000 for 2 h into 10 mM Tris•HCl pH 8.0 to remove any remaining ammonium sulfate. IMX-DogTag was then subjected to anion exchange chromatography on an ÄKTA pure 25 fast protein liquid chromatography (FPLC) system (GE Healthcare). The protein was loaded onto a 1 mL quaternary high performance (Q-HP) column (GE Healthcare) at 0.5 mL/min and eluted with 10 mM Tris•HCl, 180 mM NaCl pH 8.0. Eluted protein was incubated for 2.5 h with 2 mM diamide (Sigma) at 4 °C followed by three times 1,000-fold dialysis into 50 mM Tris•HCl pH 8.0. Concentrations of all proteins was determined using the Pierce bicinchoninic acid (BCA) Assay Kit (Thermo Fisher Scientific). The concentration of IMX-DogTag refers to the concentration of the individual subunits.

### Expression and purification of SnoopLigase

*E*. *coli* BL21 (DE3) RIPL (Agilent) were transformed with pET28a-AviTag-SnoopLigase, pET28a-SnoopLigase, or pET28a-SnoopLigaseΔC. Cells were grown on LB-Agar plates with 50 µg/mL kanamycin for 16 h at 37 °C. Single colonies were picked and grown in LB with 50 µg/mL kanamycin for 16 h at 37 °C, 200 rpm. The starter cultures were diluted 1:100 in LB with 0.8% (w/v) glucose and 50 µg/mL kanamycin and grown at 37 °C at 200 rpm until A_600_ reached 0.5. The cultures were induced with 0.42 mM IPTG and grown for 4 h at 30 °C, 200 rpm. The proteins were purified using Ni-NTA resin (Qiagen) as described^40^. SnoopLigase was dialyzed three times 1,000-fold into 50 mM sodium borate pH 10.0. AviTag-SnoopLigase was dialyzed into 50 mM Tris•borate pH 8.0. Biotinylation of AviTag-SnoopLigase was performed as previously described^75^. The His_6_-tag was cleaved from SnoopLigaseΔC using 1:50 A_280_ equivalents of MBP-TEV protease. The TEV protease digestion was performed in 50 mM sodium borate pH 10.0 with 0.5 mM EDTA for 6 h at 30 °C. The digestion reaction was then dialyzed three times at 4 °C against 1,000-fold excess 50 mM sodium borate pH 10.0 with a 3.5 kDa cut-off membrane (Fisher Scientific) and applied to Ni-NTA resin (equilibrated with 50 mM sodium borate pH 10.0) to capture the uncleaved protein, the His_6_-TEV protease and the cleaved His_6_-tag. The flow-through was concentrated and applied to a HiLoad 16/600 Superdex 75 pg column (GE Healthcare) equilibrated with 50 mM sodium borate pH 10.0. The fractions of interest were pooled, concentrated and stored at −80 °C.

### Expression and purification of CIDR

*E*. *coli* SHuffle T7 Express *lysY* (NEB, Cat. No. C3030H) were transformed with pET15b-SnoopTagJr-CIDR(ITVar07) or pET15b-CIDR(IT4var07) and grown on LB-Agar plates with 100 µg/mL ampicillin for 16 h at 37 °C. Individual colonies were picked and grown in 2 × TY with 0.8% (w/v) glucose with 100 µg/mL ampicillin for 16 h at 37 °C, 200 rpm. The starter cultures were diluted 1:100 in 2 × TY with 0.8% (w/v) glucose and 100 µg/mL ampicillin and grown at 37 °C at 200 rpm until A_600_ reached 0.7. The cultures were induced with 0.42 mM IPTG and grown for 14 h at 22 °C at 200 rpm. Cells were lysed in 50 mM Tris•HCl, 300 mM NaCl, 15 mM imidazole, pH 8.0, supplemented with 0.5% (v/v) Triton X-100, 10% (v/v) glycerol, 1 mg/mL lysozyme, 2 U/mL benzonase, PMSF, and EDTA-free mixed protease inhibitors. The proteins were purified using Ni-NTA resin as described^55^. His_6_-tag was cleaved from SnoopTagJr-CIDR and CIDR using 1:100 A_280_ equivalents of MBP-TEV protease. The TEV protease digestion was performed in 50 mM Tris•HCl, 300 mM NaCl, pH 8.0 (SnoopTagJr-CIDR) or 50 mM Tris•HCl, 150 mM NaCl, pH 9.3 (CIDR) with 0.5 mM EDTA for 4 h at 30 °C. The digestion reaction was then dialyzed three times at 4 °C against 1,000-fold excess 50 mM Tris•HCl, 300 mM NaCl, pH 8.0 (SnoopTagJr-CIDR) or 50 mM Tris•HCl, 150 mM NaCl, pH 9.3 (CIDR) with a 3.5 kDa cut-off membrane (Fisher Scientific) and applied to Ni-NTA resin to capture uncleaved protein, His_6_-TEV protease and cleaved His_6_-tag. The flow-through was further purified by size-exclusion chromatography on a HiLoad 16/600 Superdex 75 pg column (SnoopTagJr-CIDR) or HiLoad 16/600 Superdex 200 pg column (CIDR) (GE Healthcare). The proteins were eluted with 20 mM HEPES, 500 mM NaCl, pH 7.5 (SnoopTagJr-CIDR) or 25 mM Tris•HCl, 150 mM NaCl, pH 8.5 (CIDR).

### Expression and purification of CyRPA

Suspension Expi293HEK cells (Thermo Fisher Scientific) were cultured in Expi293 expression media (Thermo Fisher) at 110–130 rpm in a humidified Multitron cell incubator (Infors HT) at 37 °C with 7% CO_2_. Cells were transfected transiently using ExpiFectamine 293 transfection reagent (Thermo Fisher Scientific) with either pENTR4-LPTOS-CyRPA-SnoopTagJr or pENTR4-LPTOS-CyRPA. Transfection enhancers (Thermo Fisher) were added 16–18 h after transfection. Cell supernatants were harvested 4 days post transfection, filtered through 0.45 μm syringe filters and the proteins were purified using Capture Select C-tag Affinity Matrix (Thermo Fisher Scientific)^55^ followed by size exclusion chromatography on a HiLoad 16/600 Superdex 200 pg column (GE Healthcare). The proteins were eluted with 50 mM Tris•borate pH 7.25.

### Peptide reactivity

The SnoopTagJr-Telo peptide (GKLGSIEFIKVNKGEARPALLTSRLRFIPK) was synthesized by Insight Biotechnology at >95% purity, validated by HPLC and mass spectrometry. SnoopTagJr-Telo is SnoopTagJr linked to a 16 amino acid human telomerase reverse transcriptase mutant epitope (EARPALLTSRLRFIPK). SnoopTagJr-Telo was dissolved at 1.47 mg/mL in a 2:3 mixture of H_2_O:acetonitrile. IMX-DogTag at 5 μM was incubated with 2× molar excess of biotinylated SnoopLigase and 2× molar excess of SnoopTagJr-Telo in 50 mM Tris•borate pH 8.0 + 15% (v/v) glycerol at 4 °C for 48 h. Peptide diffuses rapidly out of polyacrylamide gels, so was barely detected upon Coomassie staining.

### SDS-PAGE

SDS-PAGE was performed on 12, 16 or 18% Tris-glycine gels using an XCell SureLock system (Life Technologies). Samples were mixed with 6× SDS loading buffer to a final concentration of 1× and heated for 3 min at 99 °C. For reduced gels 100 mM 2-mercaptoethanol was included in the SDS loading buffer.

Gels were stained with InstantBlue Coomassie stain (Expedeon) and imaged on a ChemiDoc XRS imager and QuantityOne (version 4.6) software (Bio-Rad). “% IMX-DogTag reacted” was calculated from band intensities as 100 × (1 − [substrate after reaction]/[substrate before reaction]).

### Dynamic light scattering

IMX-DogTag preparations in 50 mM Tris•HCl pH 9.25 were centrifuged for 30 min at 16,900 *g* at 4 °C to remove any aggregates. Samples were added to a reusable cuvette and placed in the chamber of an Omnisizer (Software OmniSIZE version 3.0, Viscotek GmbH). Measurements were performed at concentrations that gave stable readings (0.2–0.5 mg/mL). The size distribution of each sample was measured 10 times with a 10 s run duration at 20 °C. Intensity distributions were plotted with standard deviations.

### Preparation of immunogens

IMX-DogTag at 5 μM was incubated with 2× molar excess of SnoopLigase (with or without biotin) and 1.5× molar excess of SnoopTagJr-CIDR or CyRPA-SnoopTagJr in 50 mM Tris•borate pH 7.25 (for SnoopTagJr-CIDR) or pH 8.75 (for CyRPA-SnoopTagJr to enhance solubility) + 15% (v/v) glycerol at 4 °C for 48 h. To remove biotin-SnoopLigase from IMX-DogTag:antigen conjugate, reactions were incubated with washed and equilibrated HiCap streptavidin-agarose (Thermo Fisher) for 1 h at 25 °C on a tube rotor. The resin was transferred to a poly-prep column (Bio-Rad) and centrifuged at 300 *g* for 1 min. The resin was washed five times with Tris-phosphate pH 7.0 (25 mM phosphoric acid adjusted to pH 7.0 with Tris base) with 1 M imidazole pH 7.0 (adjusted with HCl) and 0.01% (v/v) Tween 20 at 25 °C. To remove residual liquid, the resin was centrifuged at 300 *g* for 1 min. To elute the product, the resin was incubated with Tris-phosphate pH 7.0 with 3.5 M imidazole pH 7.0 for 5 min at 25 °C on a Thermomixer comfort (Eppendorf) at 800 rpm to elute the product, followed by centrifugation at 300 *g* for 1 min at 25 °C. The elution step was repeated twice. The elution product was diluted to 2.1 M final imidazole concentration with MilliQ and incubated for 30 min with washed and equilibrated HiCap streptavidin-agarose (Thermo Fisher) at 25 °C to remove any remaining biotin-SnoopLigase. The eluted conjugates were dialyzed 1,000-fold three times against 50 mM Tris•HCl pH 9.25. Endotoxin levels were determined with Pierce LAL Chromogenic Endotoxin Quantitation Kit (Thermo Fisher Scientific) following the manufacturer’s instructions and were below 10 Endotoxin Units/mL for IMX-DogTag preparations, SnoopTagJr-CIDR, CyRPA-SnoopTagJr, and CyRPA. Endotoxin depletion of the purified IMX-DogTag sample was performed by Triton X-114 phase separation as described previously^78^.

### Immunization

All animal experiments and procedures were performed according to the UK Animals (Scientific Procedures) Act Project License (PPL PA7D20B85) and approved by the University of Oxford Animal Welfare and Ethical Review Body. Age-matched 8–10 weeks old female BALB/c mice (Envigo), housed in specific-pathogen free environments, were immunized with equal amounts of vaccines intra-muscularly into each leg. The intra-muscular route was chosen as it is the most used route for human vaccination and often causes less injection site reaction compared to subcutaneous immunization^79–81^. Immunizations were performed as prime double-boost regimens (CIDR: prime on day 0, boost on day 13 and 32;^25,82,83^ CyRPA: prime on day 0, boost on day 19 and 35). The vaccines were formulated 1:1 in the adjuvant AddaVax (InvivoGen) (25 μL/per dose). Vaccine doses were normalized to protein antigen content and each mouse was immunized with 1 μg antigen. The AddaVax adjuvant and the vaccines were mixed by pipetting prior to injection. Blood samples were harvested to obtain sera for analysis of endpoint ELISA titers (CIDR and CyRPA). Blood samples were left to clot over night at 4 °C and sera were transferred to fresh microcentrifuge tubes after 10 min centrifugation at 8,600 *g* in a bench top centrifuge. 6 mice were used for each condition.

### Endpoint ELISA

MaxiSorp plates (Thermo Fisher Scientific) were coated overnight at 4 °C with CIDR (IT4var07), CyRPA, IMX-DogTag, or SnoopLigaseΔC at 1 μg/mL in coating buffer (15 mM sodium carbonate with 35 mM sodium bicarbonate, pH 9.6). The His_6_-tag of CIDR (IT4var07) and SnoopLigaseΔC had been removed using MBP-TEV protease. IMX-DogTag had no purification tag, while CyRPA has a C-tag. Plates were washed six times with PBS/T (PBS with 0.5% Tween 20) and blocked with PBS/T with 10% skim milk for 1 h at 25 °C. Plates were washed as previously with PBS/T and incubated with duplicates of 3-fold serially diluted serum samples for 2 h at 25 °C. Following a wash step as before with PBS/T, goat anti-mouse total IgG conjugated to alkaline phosphatase (Sigma-Aldrich) (1:3,000 dilution in PBS/T) was added to the plate and incubated for 1 h at 25 °C. After a final wash step with PBS/T, p-nitrophenylphosphate (Sigma-Aldrich) at 1 mg/mL diluted in diethanolamine buffer (1 M diethanolamine, pH 9.8, Thermo Scientific) was used as a developing substrate. A_405_ was obtained using a SpectraMAX M3 plate-reader (Molecular Devices). The endpoint titer is defined as the x-axis intercept of the dilution curve at an absorbance value greater than the mean A_405_ plus five standard deviations for a serum sample from a naïve mouse at a serum dilution of 1:100.

### Lyophilization and reactivity of lyophilized protein

30 µL aliquots of IMX-DogTag at 10 μM in 50 mM Tris•borate pH 7.25 were prepared in 100 μL thin-wall PCR tubes (StarLab). The tubes were snap-frozen in a dry ice/ethanol bath for 30 min. Lyophilization was performed in a BenchTop 2K freeze-dryer (VirTis) for 48 h under vacuum at 0.14 mbar and −72.5 °C. Samples were then stored at 37 °C in a glass scintillation vial sealed with Parafilm (Sigma) on a bed of Drierite (Sigma-Aldrich). Aliquots were removed at different time points to analyze conjugation efficiency. Samples were reconstituted in reaction buffer to give a final concentration of 10 μM of all protein components and 50 mM Tris•borate pH 7.25 + 15% (v/v) glycerol. Reactions were performed for 2 h at 4 °C and analyzed by SDS-PAGE and densitometry.

### Thermal resilience analysis

IMX-DogTag at 50 μM in 50 mM Tris•borate pH 7.25 was incubated at 4–99 °C for 2 h using a C1000 thermal cycler (Bio-Rad) and then cooled to 12 °C for 30 min. Heat-treated IMX-DogTag was used to test protein conjugation efficiency with SnoopLigase and SnoopTagJr-AffiHER2 in 50 mM Tris•borate pH 7.25 + 15% (v/v) glycerol at 4 °C for 2 h. Reaction was analyzed by SDS-PAGE with Coomassie staining and densitometry.

### Statistical analysis

Statistical analysis between immunization groups was performed using GraphPad Prism (version 7.0d). Comparisons between more than two groups were made using a Kruskal–Wallis test. Dunn’s multiple comparison post-test was performed for significant values.

Further information and request for resources and reagents should be directed to and will be fulfilled by the Lead Contact, Mark Howarth (mark.howarth@bioch.ox.ac.uk).

## Supplementary information

Supp Figures
