# Supplementary material for: SnoopLigase peptide-peptide conjugation enables modular vaccine assembly
Source: Sci Rep. 2019 Mar 15;9:4625. doi: 10.1038/s41598-019-40985-w (PMC6420506; doi:10.1038/s41598-019-40985-w)
Supplement: Supplementary file 1 — Supp Figures [file 41598_2019_40985_MOESM1_ESM.pdf]

Supplementary information:

SnoopLigase peptide-peptide conjugation enables modular vaccine assembly

Anne-Marie C. Andersson[1], Can M. Buldun[1], David J. Pattinson[2], Simon J. Draper[2] and Mark Howarth[1]*.
[1]Department of Biochemistry, University of Oxford, South Parks Road, Oxford, OX1 3QU, UK. [2]Jenner Institute, University of Oxford, Oxford, OX3 7DQ, UK.

*Correspondence and requests for materials should be addressed to M.H. (email: mark.howarth@bioch.ox.ac.uk)

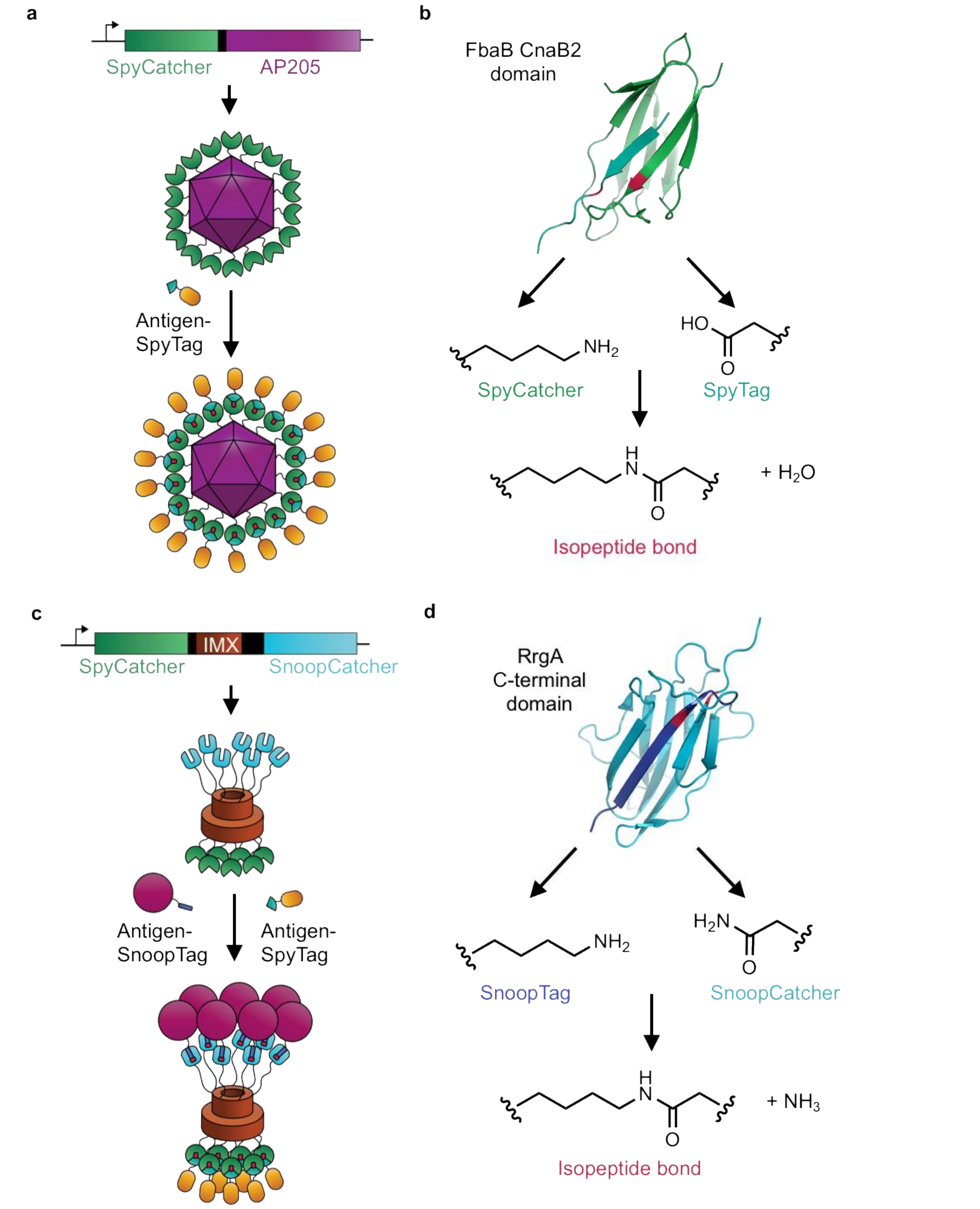

Supplementary Figure S1. Previous approaches for Plug-and-Display nanoparticle decoration. (**a**) Plug-and-Display on the bacteriophage-derived AP205 virus-like-particle by genetic fusion of SpyCatcher to the N-terminus of the AP205 coat protein and spontaneous reaction with Antigen-SpyTag. (**b**) SpyTag (turquoise) and SpyCatcher (light green) were derived from splitting of the CnaB2 domain and react together by spontaneous amidation. (**c**) Dual Plug-and-Display by genetic fusion of SpyCatcher and SnoopCatcher to IMX, allowing oligomerization of two antigens. (**d**) SnoopTag (dark blue) and SnoopCatcher (cyan) were derived from splitting a domain from RrgA and react together by spontaneous transamidation.

a IMX-DogTag:
MGSSKKQGDADVCGEVAYIQSVVSDCHVPTAELRTLLEIRKLFLEIQKLKVELQGLSKE
GSGSGEGSGDI**PATYEFT**DGKHYITNEPIPPK*

b SnoopLigase:
MGSWSHHHHHHSSGGSGVNKNDKKPLRGAVFSLQKQHPDYPDIYGAIDQNGTYQNV
RTGEDGKLTFKNLSDGKYRLFENSEPPGYKPVQNKPIVAFQIVNGEVRDVTSIVPPGVP
ATYEFT*

c SnoopLigaseΔC:
MGSWSHHHHHHSSENLYFQGGSGVNKNDKKPLRGAVFSLQKQHPDYPDIYGAIDQN
GTYQNVRTGEDGKLTFKNLSDGKYRLFENSEPPGYKPVQNKPIVAFQIVNGEVRDVTSI
VPPGV*

d SnoopTagJr:
KLGSIEFIKVNK

Supplementary Figure S2. Amino acid sequences for key constructs. (**a**) IMX-DogTag sequence, showing IMX (brown), spacer (underlined) and DogTag (orange) parts. The DogTag sequence overlapping with the SnoopLigase sequence is shown in bold. (**b**) SnoopLigase sequence, showing the His-tag (cyan) and the spacer (underlined). (**c**) SnoopLigaseΔC sequence, showing the His-tag (cyan), the TEV protease site (green), and the spacer (underlined). (**d**) SnoopTagJr.

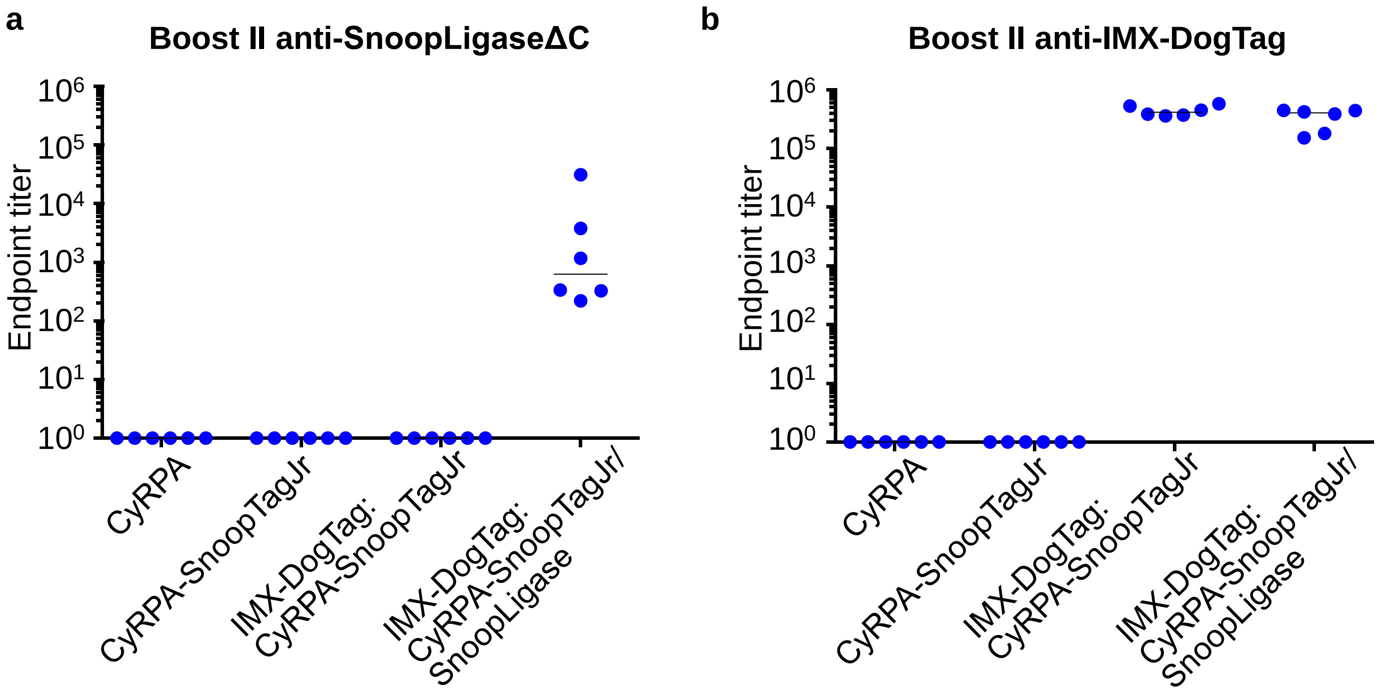

Supplementary Figure S3. Immune responses against the platform. Mice immunized against CyRPA as in Figure 4B were analyzed by ELISA for day 47 IgG responses against SnoopLigaseΔC (**a**) or IMX-DogTag (**b**). Each dot represents an individual mouse. The horizontal line is the median.
